# Alterative effects of an oral alginate extract on experimental rabbit osteoarthritis

**DOI:** 10.1186/s12929-015-0169-4

**Published:** 2015-08-04

**Authors:** Hsien-Tseng Lu, Ming-Shium Hsieh, Chao-Wen Cheng, Li-Fan Yao, Tsuey-Ying Hsu, Jai Lan, Kwang Yoon Kim, Suk Jung Oh, Yung-Hsiang Chang, Chian-Her Lee, Yung-Feng Lin, Chien-Ho Chen

**Affiliations:** Graduate Institute of Clinical Medicine, College of Medicine, Taipei Medical University, Taipei, Taiwan; Department of Orthopedics, Taipei Medical University Hospital, Taipei, Taiwan; Department of Orthopedics, En Chu Kong Hospital, New Taipei, Taiwan; School of Medical Laboratory Science and Biotechnology, College of Medical Science and Technology, Taipei Medical University, 250 Wu Xing St., Taipei, 110 Taiwan; Mastervet International Marketing Limited, Taipei, Taiwan; Ecobio Inc., Nonsan, Chungbuk Korea; School of Medicine, College of Medicine, Taipei Medical University, Taipei, Taiwan

**Keywords:** Osteoarthritis, Anterior cruciate ligament transection, Interleukin-1β, Matrix metalloproteinases, Aggrecan, CTX

## Abstract

**Background:**

Osteoarthritis (OA) is a common joint disease that causes disabilities in elderly. However, few agents with high efficacy and low side effects have been developed to treat OA. In this study, we evaluated the effects of the alginate extract named CTX in OA cell and rabbit models.

**Results:**

CTX was formulated by hydrolyzing sodium alginate polymers with alginate lyase and then mixing with pectin. HPLC was used to analyze the CTX content. Human chondrosarcoma SW1353 cells treated with interleukin-1β were used as OA model cells to investigate the effects of CTX on chondrocyte inflammation and anabolism. CTX at concentrations up to 1000 μg/ml exerted low cytotoxicity. It inhibited the gene expression of proinflammatory matrix metalloproteinases (MMPs) including MMP1, MMP3 and MMP13 in a dose-dependent manner and increased the mRNA level of aggrecan, the major proteoglycan in articular cartilage, at 1000 μg/ml. Thirteen-week-old New Zealand White rabbits underwent a surgical anterior cruciate ligament transection and were orally treated with normal saline, glucosamine or CTX for up to 7 weeks. Examinations of the rabbit femur and tibia samples demonstrated that the rabbits taking oral CTX at a dosage of 30 mg/kg/day suffered lesser degrees of articular stiffness and histological cartilage damage than the control rabbits.

**Conclusions:**

The gene expression profiles in the cell and the examinations done on the rabbit cartilage suggest that the alginate extract CTX is a pharmaco-therapeutic agent applicable for OA therapy.

## Background

Osteoarthritis (OA) is a gradually progressing disorder affecting mammalian joints in which the articular cartilage and the surrounding extracellular matrix (ECM) are destroyed [1, 2]. An imbalance between the repair and degradation of the cartilage may disrupt the collagen matrix, resulting in OA. The pathologic changes include proteoglycan degradation, type II collagen degradation, and eventually local or complete loss of the cartilage matrix [3]. Cytokines and their downstream targets are major players in the pathogenesis of OA [4, 5]. Pro-inflammatory cytokines such as interleukin (IL)-1β are produced by activated synoviocytes and articular chondrocytes and promotes the expression of several matrix metalloproteinases (MMPs), including MMP-1, MMP-3, and MMP-13 [6–8]. Many studies demonstrated that chondrosarcoma SW1353 cells challenged with IL-1β show similarities to primary human osteoarthritic chondrocytes [8–11]. IL-1β induces nuclear factor κ-B (NFκB) as a common transcriptional regulator resulting in a strong induction of those MMPs and the other cytokine IL-6 in both SW1353 cells and primary human chondrocytes. IL-1β-treated SW1353 cells can be of value to serve as a model for OA.

The development of OA therapeutics focuses primarily on disease-modifying OA drugs (DMOADs) and connective tissue structure-modifying agents (CTSMAs) [12–15]. Our team as well as others showed OA-relieving effects of injectable hyaluronan, a polysaccharide and major component of the cartilage, and suggested it as a long-lasting therapeutic agent for OA [10, 16, 17]. A noninvasive dietary supplement of glucosamine has been used to treat OA and is available clinically in some areas; however, it may increase the risk of developing diabetes with high dosages in long-term therapy [18]. Another polysaccharide, alginate, is a potential OA therapeutic agent which has also been studied in the form of injectable hydrogels in cartilage regeneration [19, 20]. It would be worth investigating the effects of orally administered alginate.

Alginate is a family of natural polysaccharides distributed in the cell walls of algae. It has a great potential for use in biomedical applications, especially in tissue engineering because of its non-toxic nature, gentle sol/gel transition procedure and low cost [21–24]. Moreover, alginate oligosaccharides produced by enzymatic degradation of alginate polymers are also known to have several biological activities including suppression of fibroblast proliferation and collagen synthesis in human skin, stimulation of endothelial cell growth and migration, stimulation of human keratinocyte growth, and suppression of Th2 development and IgE secretion [22, 25, 26]. Results suggest that alginate is useful for the treatment of disorders related to abnormal collagen metabolism such as OA.

Recent studies suggest that OA progression is associated with biomarkers of synthesis, degradation and inflammation of collagen [27]; however, these markers are usually nonspecific to OA. The severity of OA is clinically estimated by radiation or magnetic resonance imaging. To show pathological evidence on experimental animals, macroscopic and histologic examinations are mostly used.

In this study, we hypothesize that the alginate extract named CTX administered orally exerts alterative effects in OA model cells and animals.

## Methods

### Preparation of the alginate extract CTX

An alginate oligosaccharide (oligomer) solution was obtained by hydrolyzing sodium alginate polymers (~220 kDa) (Junsei Chemical Co., Japan) with alginate lyase (Sigma Adrich, U.S.A.) in distilled water at 40 °C for 24 h. CTX was formulated by mixing the alginate oligomers with pectin (Duksan Science, Korea) in a solution at 9:1 ratio and dried in a spray dryer. Alginate polymers and oligomers were determined by an HPLC (Shimadzu 10AVP series) with Supelcogel-H column (Sigma Aldrich, USA), and the procedure was modified from a protocol described elsewhere [28]. Briefly, the column temperature was 75 °C and the flow rate was 0.6 ml/min. Phosphoric acid at 0.1 % as mobile phase along with a UV detector wavelength at 210 nm were used for alginate polymer analysis; deionized water as mobile phase along with a refractive index detector were used for alginate oligomer analysis. To determine the molecular weight, the formulated CTX was analyzed by gel permeation chromatography with 0.1 M NaNO_3_ as mobile phase in Shodex Asahipak GS-320/220 columns at 35 °C.

### Cell culture

The human chondrosarcoma SW1353 cell line was obtained from the ATCC (American Type Culture Collection) [9]. SW1353 cells were cultured in L-15 medium supplemented with 10 % fetal bovine serum (FBS) and 1 % L-glutamine in a humidified incubator with 5 % CO_2_ at 37 °C. Cells at 10^5^/ml were pretreated with 0, 10, 100 or 1000 μg/ml CTX for 30 min and stimulated with 5 ng/mL recombinant human IL-1β (R&D Systems Inc.) for up to 24 h. The cells were then collected and analyzed as indicated.

### Cell viability assay

SW1353 cells were cultured in 24-well flat-bottomed tissue culture plates and treated with 0, 10, 100 or 1000 μg/ml CTX (sterilized with 0.22 μm filter) for 24 h. After incubation, the medium was replaced with 100 μl of a mixture of a ratio of 1:9 composing MTT (3-[4,5-dimethylthiazol-2-yl]-2,5-diphenyltetrazolium bromide) and medium. Cells were then incubated for 2 h and analyzed at an absorbance of 550 nm. The rate of tetrazolium reduction is proportional to the cell number.

### Reverse transcription-polymerase chain reaction (RT–PCR)

Cells were preincubated with 0, 10, 100 or 1000 μg/ml CTX for 30 min. To model OA pathogenesis, a set of cells was stimulated with 5 ng/ml IL-1β for 6 h. Total RNA was extracted (TRIzol) from chondrosarcoma cells. The RNAs (1 μg) were reversely transcribed into cDNAs using oligo-dT primers and GoScript™ Reverse Transcription System (Promega, life sciences). The presence of MMPs, Aggrecan and β-Actin mRNA in cells were analyzed by PCR and agarose gel electrophoresis as described [10]. The results of each sample were normalized to β-actin. Primer sequences are listed in Table 1.

**Table 1:** Primer sequences for RT-PCR

Primer sequence		
MMP-1		
	Forward:	5’-CCT TCT ACC CGG AAG TTG AG-3’
	Reverse:	5’-TCC GTG TAG CAC ATT CTG TC-3’
MMP-3		
	Forward:	5’-GAA AGT CTG GGA AGA GGT GAC-3’
	Reverse:	5’-AAC CGA GTC AGG TCT GTG AG-3’
MMP-13		
	Forward:	5’-GAA TTA AGG AGC ATG GCG AC-3’
	Reverse:	5’-TAA GGA GTG GCC GAA CTC AT-3’
Aggrecan		
	Forward:	5’-TGA GGA GGG CTG GAA CAA GTA CC-3’
	Reverse:	5’-GGA GGT GGT AAT TGC AGG GAA CA-3’
β-Actin		
	Forward:	5’-ACA CTG TGC CCA TCT ACG AG-3’
	Reverse:	5’-TAC AGG TCT TTG CGG ATG TC-3’

### Animals

Thirteen-week-old New Zealand White male rabbits with mature skeletons were used as the experimental OA animals. The animals were kept in steel cages (35 × 53 × 35 cm) (W × D × H) individually at 22 ± 3 °C and 55 ± 20 % humidity. Animals were fed RC4 pellet-type laboratory-animal food. There was no extra calcium supplied, and tap water was given freely. All animal procedures were approved by the Institutional Animal Care and Use Committee at Taipei Medical University.

### Experimental OA model

Rabbits were divided into five groups of ten each. Four groups were experimental OA-induced animals prepared according to the protocol of an anterior cruciate ligment transection (ACLT) [29]. The rabbits were anesthetized using a combination of Zoletil (Tiletamine + Zolezepam) (Zoletil-Virbac, Carros, France) and Rompun (Bayer, Leverkusen, Germany). Their right knee joints were incised aseptically two cm down the lateral aspect of the patella to expose and cut the anterior cruciate ligment. The subdermal muscular layer and skin was sewn by knotting absorbable and nylon sutures. Antibiotics were applied subcutaneously near the thigh.

The group that did not undergo the ACLT procedure (normal) and one group with ACLT were fed normal saline to serve as controls. The other three groups were fed either glucosamine (10 mg/kg/day) or CTX (10 or 30 mg/kg/day). All agents were administered orally from the first day of the ACLT procedure to 7 weeks.

### Pain assessment

The experimental OA rabbits were orally treated with normal saline (10 mg/kg/day), glucosamine (10 mg/kg/day) or CTX (10 or 30 mg/kg/day). Both hind paws of the rabbits were weighed the day before the surgery and weekly up to seven weeks after the surgery. The percent (%) weight distribution of the experimental right hind paw was calculated as described [17, 30].

### Specimen collection

After the rabbits were euthanized, knee joint specimens were collected by osteotomy 3 cm above and below the joints and fixed in 10 % buffered formalin (pH 7.4) for 24 h. Fixed specimens were cleared of soft tissues and ligaments allowing the gross examination of the articular surfaces of the femoral condyles and tibial plateaus.

### Macroscopic examination

The macroscopic examination of the specimens was performed using a surgical magnifying glass to evaluate the OA progression based on a modification of the parameters described previously [31] and is summarized in Table 2. The presence of fissures (V-shaped cleft), osteophytes/chondrocytes, fibrillation (surface fragmentation), cartilage ulceration (erosion), and the loss of the superficial layer were evaluated based on the location, type, and size of the pathological features. All the samples were blindly examined. Digital photographs of the articular surfaces were taken.

**Table 2:** Macroscopic scoring parameters

Articular cartilage abnormality	Score				
	0	1	2	3	4
Fissure	None	Very small	1 small	2 small or l large	3 small or 2 large
Osteophytes	None	Very small	Small	Medium	Large
Fibrillation	None	Noticeable	Moderate	Marked	Extensively severe
Ulceration	None	Mild	Moderate	Focally severe	Extensively severe
Loss of superficial layer	Normal	Slight	Moderate	Focally severe	Extensively severe

### Histological (microscopic) examination

The histological examination was performed at the femoral condyle of the rabbits. The specimens were fixed in 10 % formalin (pH 7.4) containing 0.5 % cetylpyridinium chloride and sectioned. After decalcification, the specimens were stained with hematoxyline and eosin (H&E) and Alcian blue separately. The pathological scoring parameters are listed in Table 3 and used to evaluate the severity of OA on a scale of 0 to 3. The average score of all the sites represented the OA severity of an animal.

**Table 3:** Histopathological scoring parameters

Score	Description
0	Normal matrix and chondrocytes
1	Uneven cartilage surface with loss of metachromasia around the enlarged cartilage of superficial zone. No fibrillation chondrocyte clusters
2	Some surface erosion, fibrillation and small chondrocyte clusters in superficial zone decreased metachromasia extending to the deep zone
3	Deeper surface erosion with fibrillation extending into the deep zone, large number of chondrocyte clusters containing several cells, major degenerative changes and loss of metachromasia in the cartilage matrix

### Statistical analysis

At least three independent sets of experiment were performed. Results were analyzed using SigmaPlot 12.5 software. Statistical analysis was done with Student’s *t* test and one way ANOVA. Differences were considered significant if p < 0.05 (*) or 0.01 (**).

## Results

### CTX inverted OA pathogenesis in vitro

**Fig. 1:**
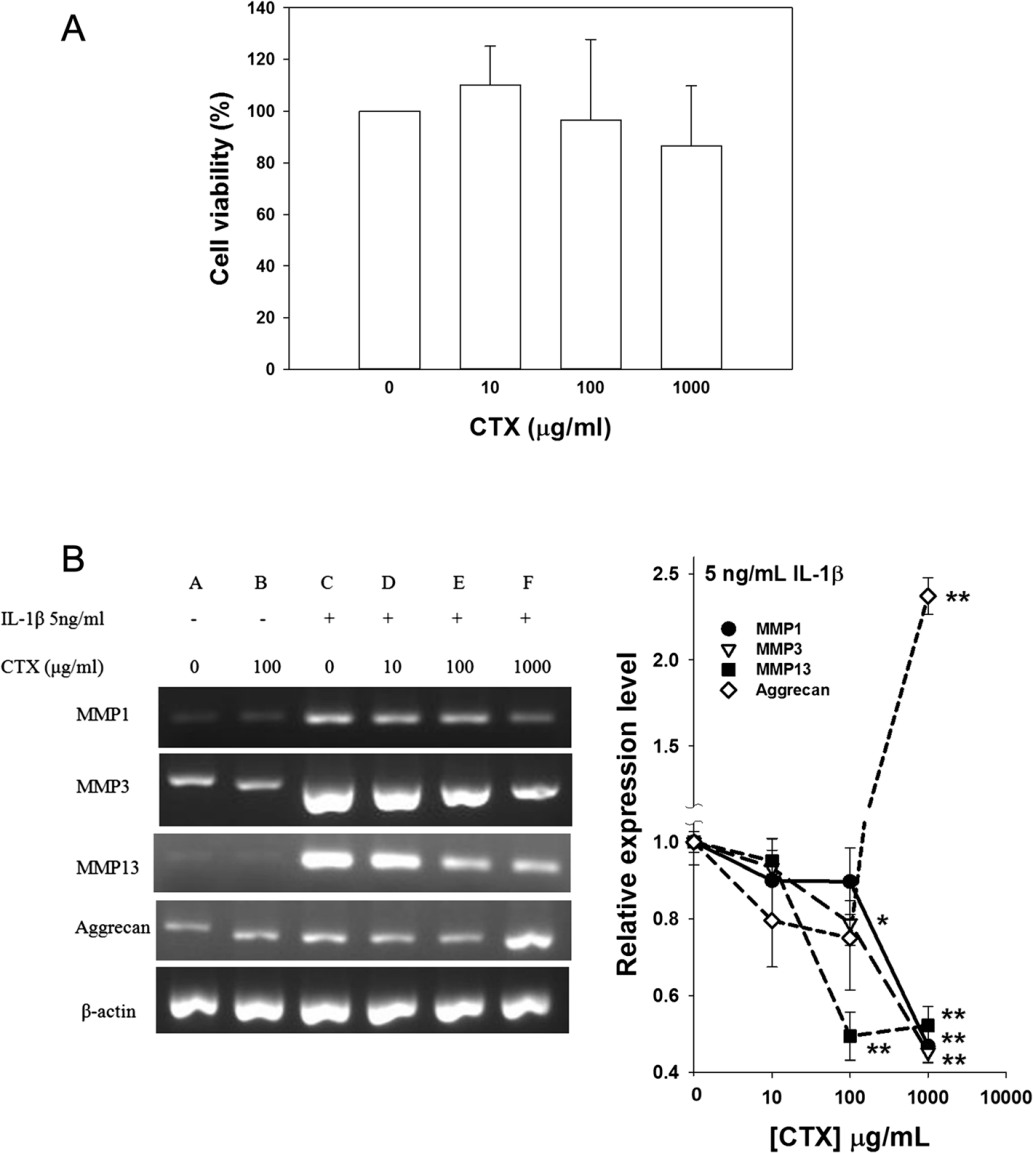
The influence of CTX on SW1353 human chondrosarcoma cells. **a** Cell survival at various concentrations of CTX. The surviving cells were detected by an MTT assay. The relative density of surviving cells was normalized to the control group, which was set as 100 %. Four independent sets of experiments were performed. **b** Effects of CTX on MMPs and aggrecan gene expression in the cells. The gene expression of MMP-1, MMP-3, MMP-13 and aggrecan were examined by RT-PCR and agarose gel electrophoresis (left panel). The density of β-actin was used as the loading control. The results from IL-1β-induced cells were plotted, and CTX-treated results were normalized to the control (right panel). Data were expressed as the mean ± SE from three independent sets of experiments. **p < 0.01

After the lyase hydrolyzation alginate polymers were converted into oligomers. The average molecular weight of the hydrolyzed product CTX was estimated 2,550 Dalton. Chondrosarcoma SW1353 cells were tested with different concentrations of the alginate extract CTX. Their viabilities were not noticeably affected by CTX at concentrations up to 1000 μg/ml in 24 h (Fig. 1a), suggesting a satisfactory safety of CTX to be investigated in cellular anabolism.

When the OA model cells were treated with CTX for 6 h, their mRNA expressions of MMP-1, MMP-3, MMP-13 and aggrecan were analyzed using RT-PCR. As expected, IL-1β induced mRNA levels of the MMPs in the cells (Fig. 1b). CTX reduced their expressions in a dose-dependent manner with a significant difference at 100 μg/ml for MMP3 and MMP13 and 1000 μg/ml for all the MMPs, suggesting an anti-inflammatory effect of the agent. Furthermore aggrecan, the major proteoglycan found in the articular cartilage [32], was dramatically increased by CTX at 1000 μg/ml, indicating a counteracting effect of CTX on OA pathogenesis.

### Animals recovered from OA with oral CTX

**Fig. 2:**
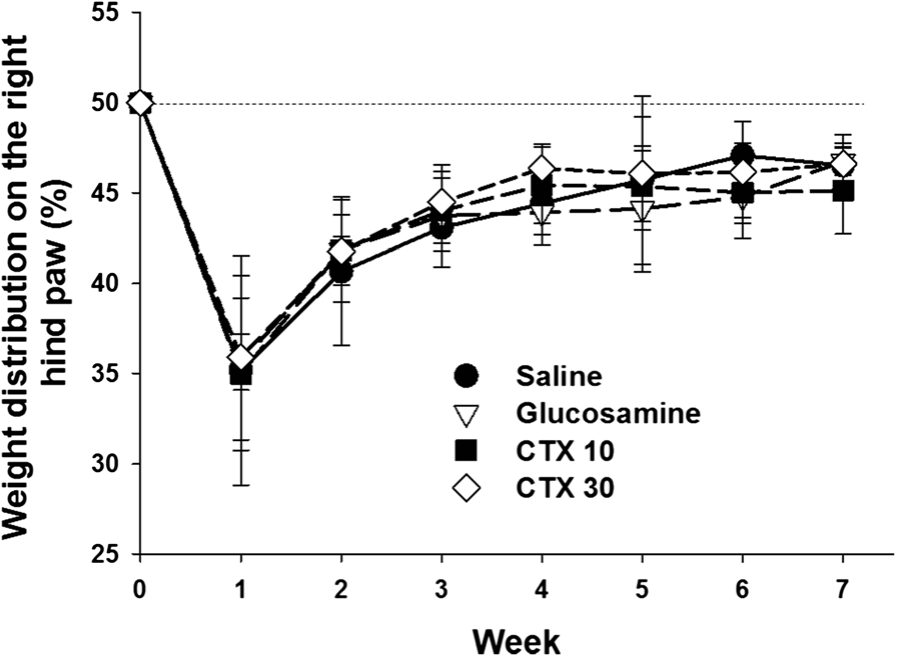
Pain assessment on experimental OA rabbits with different treatments. The rabbits were orally treated with normal saline, glucosamine or CTX as described in Methods. The percentage (%) weight distribution of the experimental right hind paw was calculated as mean ± SD from 10 animals in each group

The surgery-induced OA rabbits that underwent different treatments were monitored for pain on the experimental right hind paw. The percent weight distribution on the right hind paw was largely reduced in all the OA rabbit groups, indicating a pain on their joints (Fig. 2). The pain was relieved gradually with time. Although no treatments including glucosamine and CTX exhibited analgesic activity significantly, the tissue repair may be processed differentially among these treatments.

**Fig. 3:**
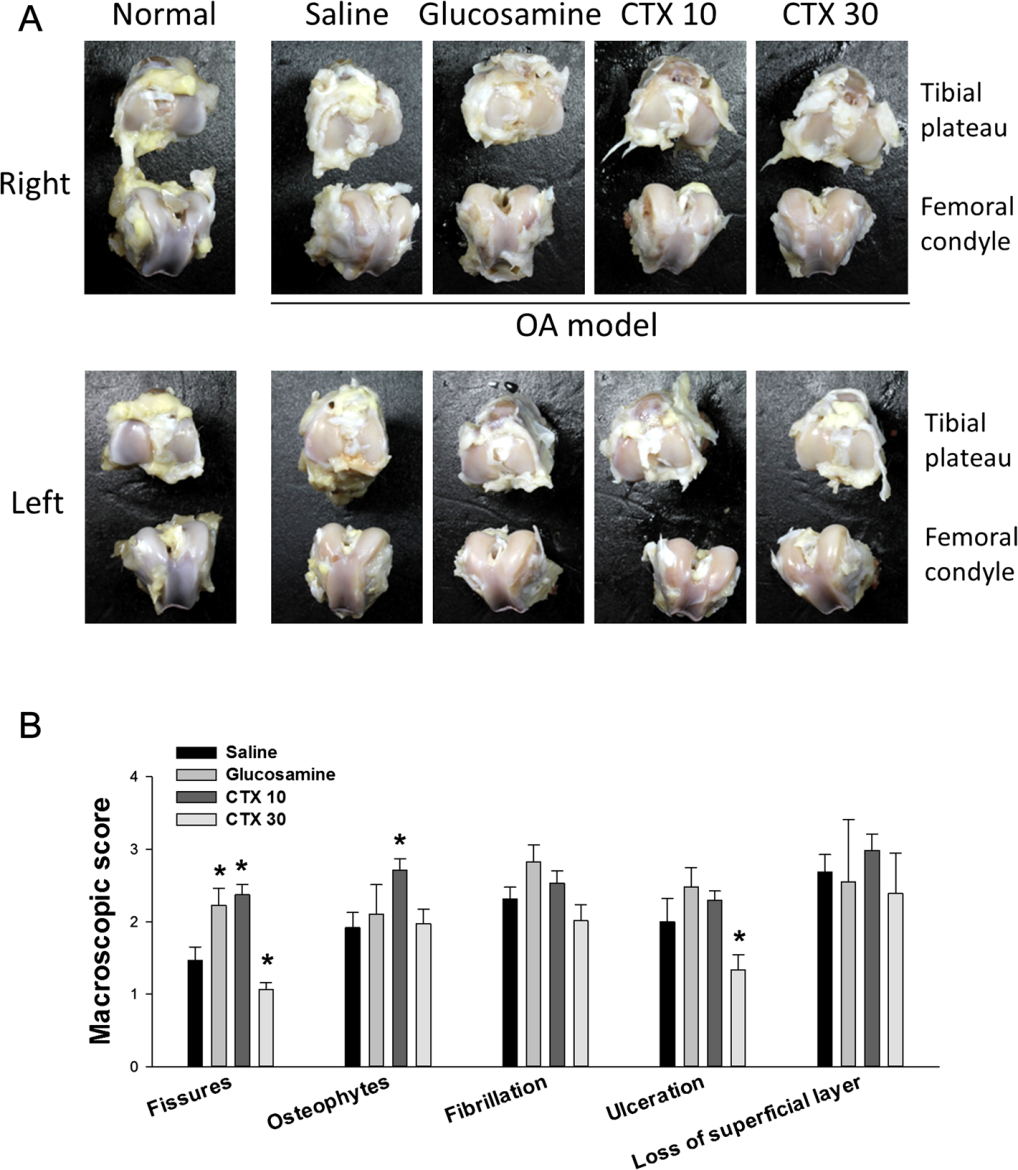
Macroscopic examination of the tibia and femur of OA rabbits. **a** The surface appearance of the tibial plateau (over) and the femoral condyle (under) of the five groups of rabbits. Normal, control (no surgery); Saline, treated 10 mg/ka/day normal saline; glucosamine, treated 10 mg/ka/day glucosamine; CTX 10, treated 10 mg/kg/day CTX; CTX 30, treated 30 mg/kg/day CTX. **b** The macroscopic appearance assessment was conducted as described in Methods and Table 2. The five evaluated items include the presence of fissure (V-shaped cleft), osteophytes/chondrocytes, fibrillation (surface fragmentation), ulceration (erosion), and loss of superficial layer. At least three animals in each group were examined. The results were compared to the group of normal saline-treated animals. * p < 0.05

The macroscopic examination of the specimens showed that the experimental OA articular cartilage was rough and dull on both femoral and tibial surfaces of the control rabbits (Fig. 3a). The most remarkable damage occurred at the femoral condyle and the tibial plateau. Glucosamine and CTX at 10 mg/kg/day were shown to be not effective. The OA groups, except for the one treated with 30 mg/kg/day CTX, displayed uneven articular surfaces with severe loss of articular cartilage. Generally, however, OA rabbits treated with CTX at 30 mg/kg/day showed improved cartilage repair with significantly lower scores of fissures and ulceration (Fig 3b). An even articular surface with regeneration of cartilaginous tissue was also observed. Thus, it is assumed that CTX inverted the progression of OA.

### OA tissue repaired with CTX treatment

**Fig. 4:**
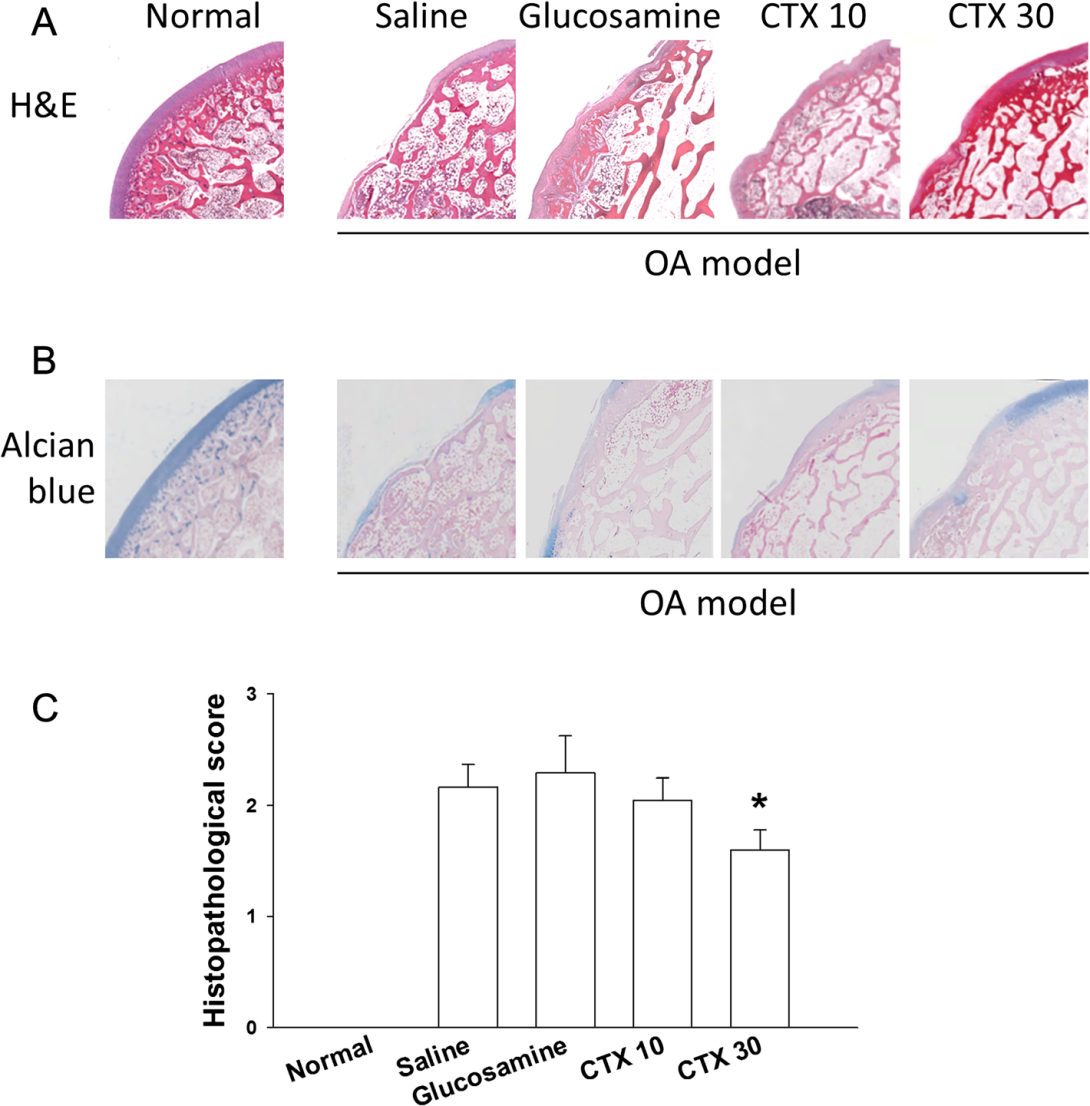
Histological examinations of cartilage at the femoral condyle. The specimens were stained with hematoxyline and eosin (H&E) (**a**) and Alcian blue (**b**) as described in Methods. Normal, control (no surgery); Saline, treated 10 mg/ka/day normal saline; glucosamine, treated 10 mg/ka/day glucosamine; CTX 10, treated 10 mg/kg/day CTX; CTX 30, treated 30 mg/kg/day CTX. **c** Histopathological scores were given as described in Methods and Table 3. The mean ± SD was calculated based on the scores of three or more animals in each group. * p < 0.05

The microscopic histological examination showed the damaged femoral condyles stained with H&E and alcian blue separately from the OA rabbits (Fig. 4a and b). There were marked cleft changes, articular cartilage proteoglycan loss, fibrosis and cleft formation with more subchondral sclerosing bone formation. Oral CTX at 30 mg/kg/day attenuated lesion formation with signs of restoration of the cartilage structure significantly (Fig. 4c). An even articular surface with regeneration of cartilaginous tissue was noticed. Overall, the treatment with CTX at 30 mg/kg/day resulted in the best improvement from OA.

## Discussion

In our study, alginate extracted CTX was suggested as a potential agent to promote matrix anabolism and stimulated cartilage regeneration. In the OA model cells, we found an effective treatment of CTX at 1000 μg/ml in reducing MMPs and promoting aggrecan expression although the effects were not obvious at low CTX (Fig. 1b). Aggrecan is important for cartilage elasticity, toughness and shock-absorbing capacity. Its degradation is a significant event in the early-stage OA [33]. Indeed, CTX improved cartilage structure restoration although it did not serve as an analgesic in the OA model rabbits (Figs. 3 and 4). Based on our data, we suggest that the CTX provide therapeutic effects for OA.

Although the etiology of OA is still intensively debated, its pathological features are well established. OA is composed by a group of overlapping distinct diseases, which may have different etiologies with similar biologic, morphologic, and clinical outcomes. The disease not only affects the articular cartilage, but also involves the entire joint, including the subchondral bone, ligaments, capsule, synovial membrane, and periarticular muscles [34]. Ultimately, the articular cartilage degenerates with fibrillation, fissures, ulceration, and loss of the joint surface. Studies showed hydrogel-based alginate possesses high viscosity and viscoelasticity which may provide good chondro-protective effects [35]. The mechanism how orally administered alginate exerts its function in articular cartilage is not known; however, our results suggest that CTX can prevent joint abrasion in early stages of OA. The digested alginate could be transformed into other glycans of the cartilage.

The group treated with 10 mg/kg/day CTX displayed an increase in fissures and osteophytes/chondrocytes (Fig. 3). This may suggest an on-going process of a feedback phenomenon, in which a greater amount of chondrocytes are recruited for the production of proteoglycan. CTX can increase aggrecan and decrease the MMPs production in chondrocytes (Fig. 1b). As erosion and loss of cartilage occurred, the remaining chondrocytes secreted more glycoprotein for bone regeneration, resulting in the production of osteophytes [17]. Thus, CTX represents an ideal DMOAD and CTSMA; however, its long-term therapeutic effects on chronic OA may need further investigation.

## Conclusions

The alginate extract CTX was found to invert OA pathogenesis in vitro by increasing aggrecan and decreasing MMPs production in the cells. It also exerted a beneficial ability *in vivo* by promoting regeneration of cartilaginous tissue. Therefore, CTX may be used in OA therapy.
